# Environmental Clonal Spread of Azole-Resistant *Candida parapsilosis* with Erg11-Y132F Mutation Causing a Large Candidemia Outbreak in a Brazilian Cancer Referral Center

**DOI:** 10.3390/jof7040259

**Published:** 2021-03-30

**Authors:** Danilo Y. Thomaz, João N. de Almeida, Odeli N. E. Sejas, Gilda M. B. Del Negro, Gabrielle O. M. H. Carvalho, Viviane M. F. Gimenes, Maria Emilia B. de Souza, Amir Arastehfar, Carlos H. Camargo, Adriana L. Motta, Flávia Rossi, David S. Perlin, Maristela P. Freire, Edson Abdala, Gil Benard

**Affiliations:** 1 Laboratory of Medical Mycology (LIM-53), Instituto de Medicina Tropical e Hospital das Clínicas da Faculdade de Medicina da Universidade de São Paulo, São Paulo 05403-000, Brazil; dan.yamamoto.thz@usp.br (D.Y.T.); gildadelnegro@gmail.com (G.M.B.D.N.); gabriellehaddad@usp.br (G.O.M.H.C.); mazo@usp.br (V.M.F.G.); 2 Central Laboratory Division (LIM-03), Hospital das Clínicas da Faculdade de Medicina da Universidade de São Paulo, São Paulo 05403-010, Brazil; adriana.motta@hc.fm.usp.br (A.L.M.); flaviarossi61@gmail.com (F.R.); 3 Center for Discovery and Innovation, Hackensack Meridian Health, Nutley, NJ 07110, USA; a.arastehfar.nl@gmail.com (A.A.); david.perlin@hmh-cdi.org (D.S.P.); 4 Cancer Institute of São Paulo State, Hospital das Clínicas da Faculdade de Medicina da Universidade de São Paulo, São Paulo 01246-000, Brazil; odeli.sejas@hc.fm.usp.br (O.N.E.S.); maria.ebsouza@hc.fm.usp.br (M.E.B.d.S.); maristela.freire@hc.fm.usp.br (M.P.F.); edson.abdala@hc.fm.usp.br (E.A.); 5 Bacteriology Center, Instituto Adolfo Lutz, São Paulo 01246-000, Brazil; carlos.pqc@gmail.com

**Keywords:** clonal outbreak, candidemia, *Candida parapsilosis*, antifungal agents, azole-resistant, drug resistance mechanisms, *ERG11* mutations, microsatellite typing, environmental reservoirs, horizontal transmission

## Abstract

Clonal outbreaks due to azole-resistant *Candida parapsilosis* (ARCP) isolates have been reported in numerous studies, but the environmental niche of such isolates has yet to be defined. Herein, we aimed to identify the environmental niche of ARCP isolates causing unremitting clonal outbreaks in an adult ICU from a Brazilian cancer referral center. *C. parapsilosis* sensu stricto isolates recovered from blood cultures, pericatheter skins, healthcare workers (HCW), and nosocomial surfaces were genotyped by multilocus microsatellite typing (MLMT). Antifungal susceptibility testing was performed by the EUCAST (European Committee for Antimicrobial Susceptibility Testing) broth microdilution reference method and *ERG11* was sequenced to determine the azole resistance mechanism. Approximately 68% of isolates were fluconazole-resistant (76/112), including pericatheter skins (3/3, 100%), blood cultures (63/70, 90%), nosocomial surfaces (6/11, 54.5%), and HCW’s hands (4/28, 14.2%). MLMT revealed five clusters: the major cluster contained 88.2% of ARCP isolates (67/76) collected from blood (57/70), bed (2/2), pericatheter skin (2/3), from carts (3/7), and HCW’s hands (3/27). ARCP isolates were associated with a higher 30 day crude mortality rate (63.8%) than non-ARCP ones (20%, *p* = 0.008), and resisted two environmental decontamination attempts using quaternary ammonium. This study for the first time identified ARCP isolates harboring the Erg11-Y132F mutation from nosocomial surfaces and HCW’s hands, which were genetically identical to ARCP blood isolates. Therefore, it is likely that persisting clonal outbreak due to ARCP isolates was fueled by environmental sources. The resistance of Y132F ARCP isolates to disinfectants, and their potential association with a high mortality rate, warrant vigilant source control using effective environmental decontamination.

## 1. Introduction

Candidemia has shown a high mortality rate and increasing incidence mainly in patients with malignancy, immune impairment, and use of invasive devices. Although diagnosis, treatment, prophylaxis, and infection control have improved, invasive infections caused by *Candida parapsilosis* are still regarded as a medical emergency for susceptible patients, including those suffering from cancer [1,2,3].

Although *Candida albicans* remains the most frequent cause of candidemia, the extensive antifungal exposure may lead to the emergence of non-albicans *Candida* species [4,5]. *C. parapsilosis* is the third leading cause of candidemia worldwide and the second cause in some Southeast Asian, Middle Eastern, European, and South American countries [6,7,8,9].

*Candida parapsilosis* is known to be susceptible to azole-class drugs; however, emerging studies have implicated the surge of clonal outbreaks as due to azole-resistant *C. parapsilosis* (ARCP) isolates in numerous countries [10,11,12,13,14,15], possibly fueled by azole overuse, which complicates the treatment of azole-naïve patients and is potentially associated with a higher mortality rate [16]. More alarming is the emergence of multidrug-resistant (MDR) *C. parapsilosis* isolates, resistant to both of the most widely used frontline antifungals, i.e., fluconazole and echinocandins [17], and the lack of effectiveness of these drugs against *C. parapsilosis* biofilms [18].

The prominent biofilm formation capacity on medical devices is thought to allow this species to not only become one of the most prevalent *Candida* species in clinical settings, but also the reason behind severe clonal outbreaks [19]. Biofilm production allowed *C. parapsilosis* to avidly adhere to plastic surfaces and survive for several weeks [20], which may have also rendered this species less susceptible to some chlorine-based disinfectants when compared to other *Candida* species [21]. Although it is hypothesized that invasive infections due to *C. parapsilosis* are mostly owing to horizontal transfer from contaminated environments [22], nonetheless, the environmental niche of ARCP isolates using the application of resolutive typing tools has yet to be determined. Identification of outbreak source is of high clinical relevance to guiding effective infection control strategies and improving the clinical outcome of patients admitted to the ICU [4].

Therefore, the scope of this study was to determine the environmental source of ARCP isolates, which has probably been the cause of persistent and increasing outbreaks among cancer patients. Unprecedentedly, we identified ARCP isolates from the nosocomial surfaces and the hands of healthcare workers (HCW), which were 100% identical to the ARCP isolates recovered from blood samples, and prove the horizontal transmission of this pathogen. Furthermore, to our knowledge, the present investigation describes the largest outbreak of ARCP candidemia in a single ICU from an adult cancer center in one year, and detected for the first time the Erg11-Y132F mutation in non-clinical isolates.

## 2. Materials and Methods

**Figure 1:**
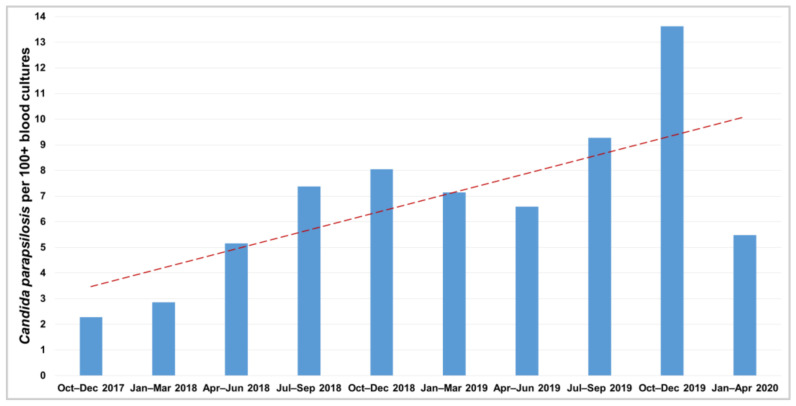
*Candida parapsilosis* isolates per 100 positive blood cultures at the cancer referral center. The red dashed trend line shows the marked increase from October 2017 to April 2020.

**Figure 2:**
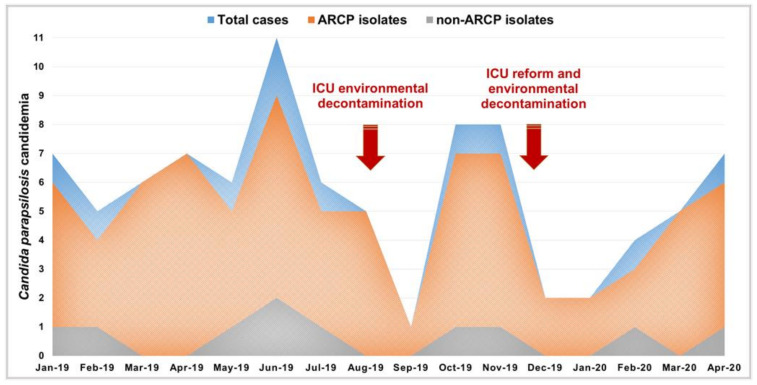
Candidemia cases by azole-resistant *C. parapsilosis* (ARCP) or non-ARCP in the adult oncology intensive care unit (ICU) from January 2019 to April 2020. The red arrows indicate the two decontamination efforts of the ICU environment with quaternary ammonium disinfectant.

**ARCP outbreak investigation.** By the end of 2018, a marked increase in the rate of *C. parapsilosis* isolates per 100 positive blood cultures compared to the end of 2017 was observed in the adult ICU from a 500-bed referral oncology center in Brazil (Figure 1). Subsequently, fluconazole (FLC) disk diffusion susceptibility testing [23] was performed on blood culture isolates at the clinical laboratory. From January to February 2019, an unexpectedly high FLC resistance rate (83.3%) was observed among the *C. parapsilosis* sensu stricto clinical isolates, highlighting a probable clonal spreading of ARCP (Figure 2).

To better characterize the ARCP outbreak in the oncology ICU, all patients from this ward were screened for ARCP in March 2019. Swab samples were collected from the axilla, groins, and the pericatheter skin according to a previously described protocol [24]. In April 2019, environmental samples were taken with sponge sticks (3 M, St. Paul, MN, USA), and the following inanimate surfaces were investigated: bedrails, cardiac monitors, ventilators, infusion pumps, sinks, and cabinets in the vicinity of the patient bed. In June 2019, samples from HCW’s hands were obtained using a standard broth plastic bag technique [25]. Swabs, sponges, and broth from plastic bags were inoculated in Brain Heart Infusion (BHI) broth with vancomycin (50 μg/mL) plus ciprofloxacin (5 μg/mL) and incubated for 72 h at 37 °C. Positive cultures were screened on chromogenic medium (ChromID Candida, bioMérieux, Marcy L’Étoile, France), and species identification was carried out by Matrix Assisted Laser Desorption Ionization Time-of-Flight (MALDI-TOF) mass spectrometry (Vitek MS, bioMérieux).

**Environmental decontamination.** The hospital’s infection prevention and control team performed a terminal cleaning of the adult oncology ICU in August 2019 and in December 2019, the latter after a partial renovation of the ward (Figure 2). First, disposable items were discarded. Movable partitions and curtains were removed and cleaned using neutral detergent and quaternary ammonium disinfectant. Then, all surfaces and equipment in the patient’s care rooms were also cleaned with the same detergent and disinfectant.

**Antifungal susceptibility testing.** The antifungal susceptibility pattern of *C. parapsilosis* sensu stricto isolates obtained from patients, HCWs, and nosocomial surfaces in 2019 were tested by the European Committee for Antimicrobial Susceptibility Testing (EUCAST) broth microdilution reference method assay according to the document E.DEF 7.3.2 [26]. All isolates were tested for in vitro susceptibility to amphotericin B (Sigma-Aldrich, St. Louis, MO, USA), anidulafungin (Sigma), micafungin (Astellas Pharma, Tokyo, Japan), FLC (Sigma), and voriconazole (VRC, Sigma). The MICs were interpreted following the EUCAST clinical breakpoints v. 10.0 [27]. Each experiment was performed at least three times on different days, and *C. parapsilosis* ATCC 22019 and *C. krusei* ATCC 6258 were used as quality control strains.

**Multilocus microsatellite typing (MLMT).** Genotyping of all *C. parapsilosis* sensu stricto isolates and the reference strain, ATCC 22019, was carried out by microsatellite analysis using PCR amplification of eight different loci with primers previously described [28]. PCR products were separated on 3% agarose gel, stained with GelRed™ (Biotium, Fremont, CA, USA), and visualized with the UVITEC gel documentation system (Cleaver Scientific, Rugby, Warks, UK). The similarity of the allelic profiles was evaluated by the Dice coefficient, and the clustering was performed by the unweighted pair group method with arithmetic mean (UPGMA) employing the Bionumerics software v. 7.6 (Applied Maths, Sint-Martens-Latem, Belgium). Cluster was defined as a group of ≥2 isolates showing an identical allelic profile [10,15].

***ERG11* gene sequencing.** DNA samples from all ARCP isolates were subjected to both PCR and sequencing of the entire open reading frame of the *ERG11* gene. PCR products were purified with the ExoSAP-IT™ (Thermo Fisher Scientific, Waltham, MA, USA) and sequenced with the 3500 Genetic Analyzer (Applied Biosystems, Foster City, CA, USA) using four specific primers previously described [29]. *ERG11* sequences were analyzed using MEGA v. X [30] and compared with the available corresponding sequence of *C. parapsilosis* ATCC 22019 (GenBank accession no. GQ302972).

**Statistical procedure.** 30-day outcome comparisons between ARCP and non-ARCP fungemia episodes were carried out using χ^2^ test. Kaplan–Meier survival curves for ARCP and non-ARCP fungemia episodes were constructed and statistical comparison of the two groups were carried out with log-rank test (GraphPad Software, San Diego, CA, USA). *p*-values of <0.05 were considered statistically significant.

## 3. Results

Antifungal susceptibility and genetic profiles were determined for 112 isolates obtained in 2019, including 73 clinical isolates (70 from blood cultures and 3 from pericatheter skins), 28 HCW isolates (27 from hands and 1 from a skin lesion), and 11 nosocomial surface isolates (7 from bedside carts, 2 from bedrails, 1 from an infusion pump, and 1 from a cardiac monitor).

**Figure 3:**
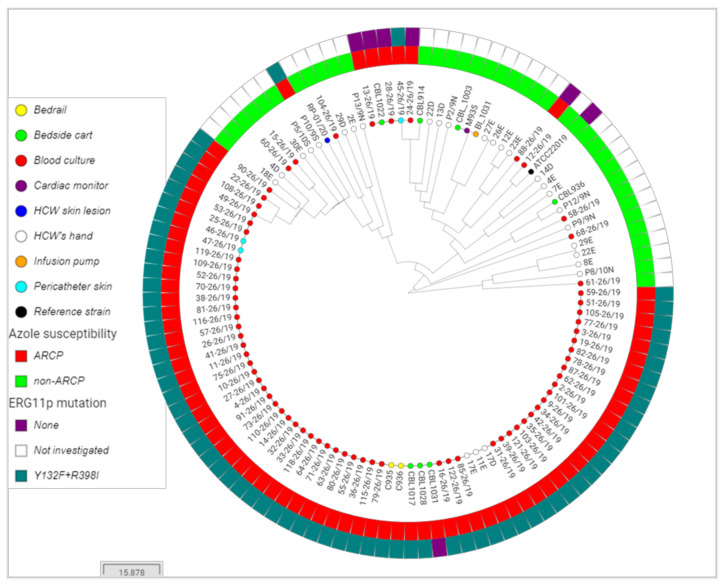
Circular tree generated from 112 *C. parapsilosis* sensu stricto isolates obtained from the adult oncology intensive care unit in 2019. The small colored circles indicate the isolate source. ARCP = azole-resistant *C. parapsilosis*.

**Figure 4:**
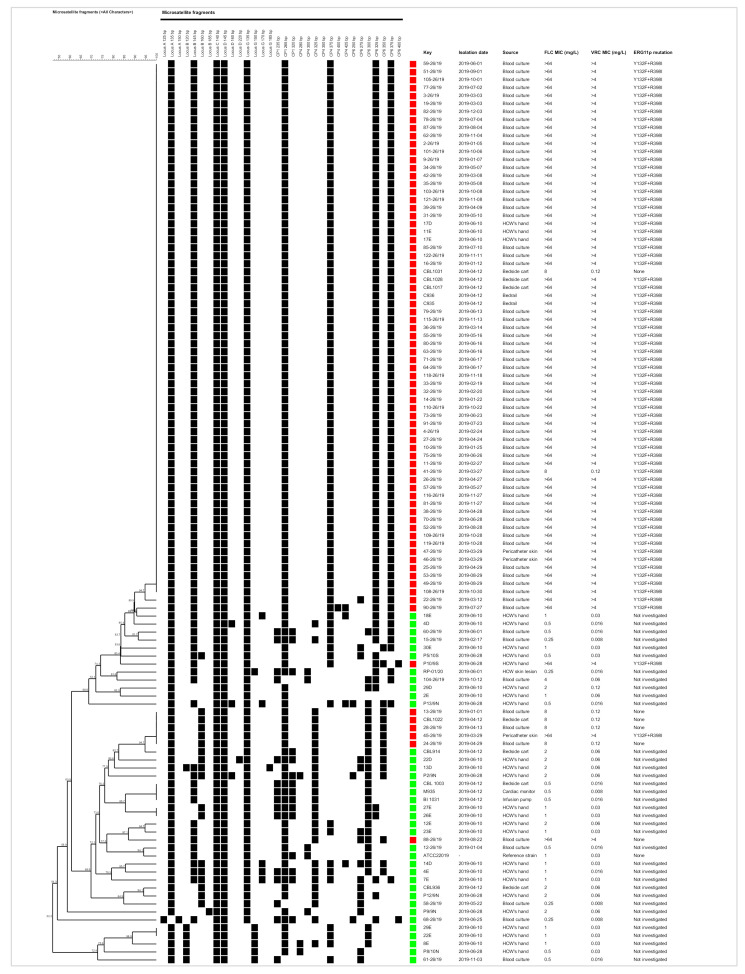
Dendrogram showing the clustering of the 112 *C. parapsilosis* sensu stricto isolates and the ATCC22019 strain based on microsatellite analysis. The black square indicates the presence of an amplification product, the red square indicates the azole-resistant isolates, and the green square indicates the non-azole-resistant ones. FLC = fluconazole, VRC = voriconazole, and MIC = minimum inhibitory concentration.

Resistance to FLC was observed in 67.9% (76/112) of the isolates: 100% (3/3) from pericatheter skins, 90% (63/70) from blood cultures, 54.5% (6/11) from nosocomial surfaces, and 14.3% (4/28) from HCWs. Seventy (92.1%) ARCP isolates harbored Erg11-Y132F + R398I mutations (Figure 3). VRC cross-resistance was observed in 92.1% (70/76) of the FLC resistant isolates (Figure 4). All isolates were susceptible to amphotericin B (0.25–1 mg/L), anidulafungin (1–2 mg/L), and micafungin (0.5–2 mg/L).

MLMT showed five *C. parapsilosis* sensu stricto clusters: three non-ARCP clusters (*n* = 3; *n* = 2; *n* = 2), and two ARCP clusters (*n* = 67; *n* = 5). The major cluster contained 88.2% (67/76) of the ARCP isolates: 100% (2/2) from beds, 81.4% (57/70) from blood cultures, 66.7% (2/3) from pericatheter skins, 42.9% (3/7) from bedside carts, and 11.1% (3/27) from HCW’s hands (Figure 3 and Figure 4).

**Figure 5:**
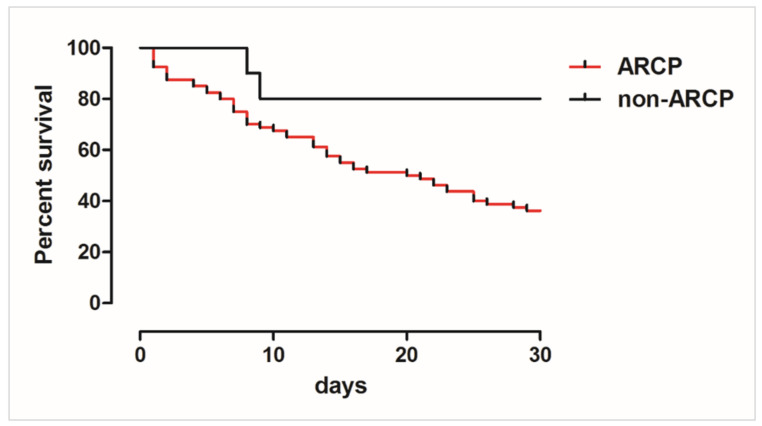
Kaplan–Meier curve for 30-day survival of candidemia patients infected with azole-resistant *C. parapsilosis* (ARCP) vs. non-ARCP (*p* = 0.025). The curve was constructed and compared with the log-rank test.

*Candida parapsilosis* isolates per 100 positive blood cultures at the end of 2018 increased 254% compared to the end of 2017 (Figure 1). Successively, ninety episodes of *C. parapsilosis* sensu stricto candidemia were detected from January 2019 to April 2020 and 88% were caused by ARCP isolates (Figure 2). These isolates were associated with higher crude mortality rate at 30 days (63.8%) than non-ARCP ones (20%, *p* = 0.008). The Kaplan–Meier curve highlighted that patients infected with ARCP isolates had a significantly higher mortality rate than those with non-ARCP (*p* = 0.025, Figure 5).

## 4. Discussion

The surging trend of *C. parapsilosis* candidemia and the high prevalence of ARCP isolates recovered from the blood samples of cancer patients suggested an outbreak with an unknown source of infection in our hospital. The comprehensive environmental sampling identified *C. parapsilosis* from various environmental sources; some of them were 100% identical ARCP blood isolates. Although environmental decontamination could initially lower the incidence of candidemia due to ARCP isolates, worryingly, the ARCP candidemia rebounded and continued to grow.

Children and low-birth-weight neonates are the patient group with increased risk for *C. parapsilosis* fungemia, possibly due to the prolonged use of parenteral nutrition and horizontal transmission from HCW’s hands [19]. Nevertheless, a recent study detected azole-susceptible *C. parapsilosis* as the most frequent candidemia agent in cancer patients, mainly adults [31]. Moreover, FLC prophylaxis and cross-infection may induce the emergence of ARCP strains in hospital settings and favor the occurrence of outbreaks [16,32]. To date, the present clonal ARCP outbreak is the largest ever detected in a single ward in a single year, and the first one documented in an adult oncology ICU.

To our knowledge, this study for the first time identified nosocomial surfaces, HCWs, and catheters as the potential source of ARCP isolates, which was evidenced using a resolutive microsatellite typing method. Since such isolates are likely associated with a higher mortality, fluconazole therapeutic failure, and resistance to decontamination efforts (Figure 2), prompt environmental screening coupled with effective infection control strategies and judicious azole use are of high value.

To identify the source of infection, we performed a comprehensive environmental screening and, similar to previous findings, we found *C. parapsilosis* as the most prevalent *Candida* isolated from the hands of HCWs and the nosocomial surfaces [33]. The presence of the same mutations in *ERG11* and identical microsatellite typing of isolates from blood cultures, pericatheter skins, HCW’s hands, and nosocomial surfaces (Figure 3 and Figure 4) strongly supports a horizontal transmission of this clone.

Although ARCP isolates have been increasingly reported [10,11,12,13,14,15,16], FLC resistance rates remain low, ranging from 2 to 5% globally [19]. In contrast, our investigation showed high FLC resistance rates in isolates from pericatheter skins (100%), blood cultures (90%), nosocomial surfaces (54.5%), and HCWs (14.3%), with VRC cross-resistance in 92.1% among them.

Similar to previous studies reporting clonal outbreaks due to ARCP, we also identified Y132F as the most prevalent mutation in Erg11 [10,11,12,13,14,16,29]. However, the additional mutation of R398I present in ARCP isolates was shown not to confer azole resistance [34]. Of note, six ARCP isolates had wild-type *ERG11* (Figure 3 and Figure 4), supporting the involvement of other molecular mechanisms, such as the overexpression of *ERG11* and efflux pumps [29]. Clonal isolates harboring Erg11-Y132F+R398I mutations were responsible for 81.4% of the 70 *C. parapsilosis* sensu stricto candidemia cases in 2019 (Figure 3 and Figure 4), resisting environmental decontamination attempts with quaternary ammonium (Figure 2), which is reminiscent of observations made for the MDR *Candida auris* [35].

Invasive candidiasis, mainly candidemia, has presented a crude mortality of 40–55% in the ICU over the last decade [4]. A previous multicenter study showed overall mortality of 35% at the 4th week in cancer patients with fungemia, with no difference among *C. albicans*, *C. glabrata*, *C. krusei*, *C. tropicalis,* and *C. parapsilosis* [36]. Worryingly, and in line with a previous study from Turkey [16], ARCP candidemia was associated with an unexpectedly much higher mortality rate (63.8%), and almost all of these episodes were caused by the clone harboring the Erg11-Y132F mutation (Figure 3 and Figure 4). The behavior of this strain is a cautionary tale for a change in the fitness of *C. parapsilosis*, usually considered one of the less virulent *Candida* species with low associated mortality rates [19,32].

## 5. Conclusions

The clonal spread of ARCP harboring theErg11-Y132F mutation in care settings and on healthcare workers was likely responsible for the persistence of this outbreak in the adult oncology ICU. Despite two attempts at environmental decontamination in 2019, the ARCP candidemia incidence remained a persistent and increasing challenge into 2020 (Figure 2). These unprecedented findings warn of breakthrough ARCP candidemia and poor outcomes in patients treated with FLC, the most widely prescribed antifungal agent in developing countries [9]. Finally, identifying azole resistance reservoirs and appropriate disinfectants to decontaminate the critical care environment are indispensable steps in attempting to interrupt the transmission of this exceptional ARCP strain carrying the Erg11-Y132F mutation.

## Data Availability

*ERG11* sequences from strains representing the two azole-resistant clusters are available at GenBank (https://www.ncbi.nlm.nih.gov/genbank/ accessed on 3 August 2020) under the accession numbers MW714301–MW714308.
